# Impact of adhesive layer addition on the optical properties of remineralized white spot lesions

**DOI:** 10.34172/joddd.025.43844

**Published:** 2025-09-30

**Authors:** Francesca Zotti, Giorgia Lanzaretti, Francesca Pilati, Nicoletta Zerman

**Affiliations:** ^1^Department of Surgical Sciences, Pediatrics and Gynecology, University of Verona, P.le L.A. Scuro 10, 37134 Verona, Italy; ^2^Private Practice, Vicenza, Italy; ^3^Private Practice, Trento, Italy

**Keywords:** Adhesive, Remineralization, Spectrophotometer, White spot

## Abstract

**Background.:**

The present in vitro study evaluated the camouflage effect of two treatments for white spot lesions (WSLs): remineralization with Mi Paste Plus© (GC Europe N.V.) and remineralization combined with a Scotchbond Multi-Purpose© (3M ESPE 2015) adhesive layer. Additionally, the study assessed the color stability of the treatments when exposed to a staining agent, such as coffee.

**Methods.:**

Sixty extracted anterior and posterior teeth were preserved in saline solution and divided into two groups. Artificial WSLs were created using 37% orthophosphoric acid for 1 minute. Both groups underwent daily 6-hour remineralization treatments with Mi Paste Plus© for 14 days. In the second group, a thin adhesive layer was applied after remineralization. Color analysis was performed using an MHT SpectroShade Micro spectrophotometer at baseline (T_1_), after remineralization (T_2_), after adhesive application for group 2 (T_R_), and after immersion in coffee for 24 hours (T_3_). Color differences (ΔE) between healthy tissue and WSLs were assessed, with ΔE≤3.3 considered clinically acceptable. Data were analyzed using STATA 17 (*P*≤0.05).

**Results.:**

ΔE analysis showed a statistically significant improvement in the camouflage effect in the adhesive-treated group (T_R_) compared to remineralization alone (T_2_). After coffee exposure (T_3_), ΔE values increased significantly in both groups, with higher values observed in the adhesive-treated group. Intragroup analyses indicated significant differences between T_2_ and T_3_, as well as T_R_ and T_3_.

**Conclusion.:**

The application of an adhesive system can be considered a valid option to improve the aesthetic outcome of a remineralization treatment; however, this approach carries the risk of resin staining over time.

## Introduction

 Dental enamel is a non-vital, densely mineralized tissue covering the anatomical crown of the tooth. It is formed by ameloblasts, which undergo involution once the anatomical crown is formed. This makes enamel an acellular tissue, incapable of regenerating or self-repairing when damaged by lesions or decay.^1,2^

 The thickness of enamel varies from approximately 2.3–2.5 mm at the level of cusps and incisal edges to about 1–1.3 mm on lateral surfaces, tapering further to a few microns near the cementoenamel junction (CEJ).

 Enamel is a highly mineralized tissue, and this important mineral content also makes it extremely susceptible to demineralization processes induced by acids produced by oral bacteria, leading to the formation of carious lesions.

 From an aesthetic perspective, several factors can affect a patient’s smile, including asymmetries, misalignments, agenesis, shape abnormalities of dental elements, or alterations in color, such as the presence of white spots.^3-5^ WSLs are areas of enamel hypomineralization without cavitation and are classified as ICDAS 1 or 2 depending on severity. With a prevalence of about 24%, WSLs most commonly occur in areas prone to plaque retention, such as the cervical third of the buccal surface or around orthodontic brackets.^6^

 White spot lesions (WSLs) appear as opaque, rough enamel areas with a chalky white color and can vary in shape and size, with more or less defined margins.

 The whitish appearance results from a loss of mineral content, with organic fluids replacing hydroxyapatite, which alters the optical behavior of enamel. In sound enamel, the refractive index (RI) is uniform (RI ≈ 1.62), allowing light to pass through unaltered to the dentinoenamel junction. In WSLs, however, the increased presence of fluids (e.g., water, RI = 1.33) introduces multiple interfaces that scatter and reflect light, producing a white appearance.

 In early stages, WSLs may only be visible after air drying, as air (RI = 1.00) further increases contrast.^6^

 Over time, numerous treatment options have been proposed to prevent WSL progression (WSLs) and improve aesthetics. Currently, composite resin restorations or veneers are considered excessively invasive, as is microabrasion, which, while achieving aesthetic improvement, involves sacrificing the superficial enamel layer.^7-9^

 For this reason, research has increasingly focused on more conservative treatments, such as erosion-infiltration techniques and remineralization therapies.

 Erosion-infiltration treatments involve the application of an infiltrating resin after etching and drying of the enamel surface.^10^ The resin used, with a penetration coefficient of 147 cm/s and a refractive index of 1.52,^7^ infiltrates the porosities via capillary action, displacing air and water. This blocks bacterial pathways, arrests the progression of the lesion, and reduces optical contrast by minimizing light scattering due to RI similarity with hydroxyapatite.^11^ These treatments reached excellent esthetic outcomes even in ICDAS 2 lesions.^6^

 Remineralization provides a biological strategy for reversing early-stage WSLs. Among the most used and studied agents is casein phosphopeptide-amorphous calcium phosphate (CPP-ACP). This complex stabilizes calcium phosphate in a bioavailable form, allowing for the controlled release of calcium and phosphate ions into the enamel. The casein component binds to enamel, plaque, and soft tissues, creating a reservoir that supports remineralization when pH drops. Calcium hydrogen phosphate (CaHPO₄), being a neutral molecule, contributes to the restoration of minerals within the enamel structure.

 CPP-ACP also reduces bacterial adhesion by modifying the salivary pellicle. When fluoride is added to the complex, it becomes stabilized and is gradually released, facilitating the formation of fluorapatite. Studies confirm that the combination of CPP-ACP and fluoride improves the surface microhardness of WSLs and provides long-term stability.^12^

 In cases where remineralization alone fails to achieve satisfactory aesthetic outcomes, an adjunctive application of a thin layer may improve optical masking. This combined strategy supports the therapeutic action of remineralizing agents while enhancing the visual integration of the lesion with the surrounding enamel.^13^

 The aims of this in vitro study were as follows:

- To evaluate, through spectrophotometric analysis, the difference in terms of masking effectiveness between two treatment methods for WSLs: remineralization treatment alone using Mi Paste Plus© (GC Europe N.V.) and remineralization treatment combined with a thin layer of adhesive Scotchbond Multi-Purpose© (3M ESPE 2015).
- To assess the color stability over time of both treatments when exposed to a staining substance, such as coffee.

## Methods

 The study was approved by the Ethics Committee of South Est Veneto (approval number: 430CET).

 The sample size determination was calculated using the statistical software G-Power v. 3.1 (University of Düsseldorf, Düsseldorf, Germany), assuming a mean ± SD ΔE value of 10.89 ± 2.95 in the control group (remineralization alone), with α = 0.05 and a statistical power of 95%. Based on these parameters, a sample size of 30 teeth per group allows for the detection of a ΔE difference of 2.79 between the treated group and the control group (effect size = 0.95). This difference is considered clinically relevant according to the literature.^14,15^

 Sixty teeth, including both anterior and posterior elements from both arches, were collected. These teeth had been extracted for periodontal reasons or removed due to trauma. Residual periodontal ligament was removed using curettes, and the teeth were cleaned with 10% hydrogen peroxide for 10 seconds, rinsed with denatured alcohol for 30 seconds, and stored in saline solution to prevent dehydration.

**Figure 1:**
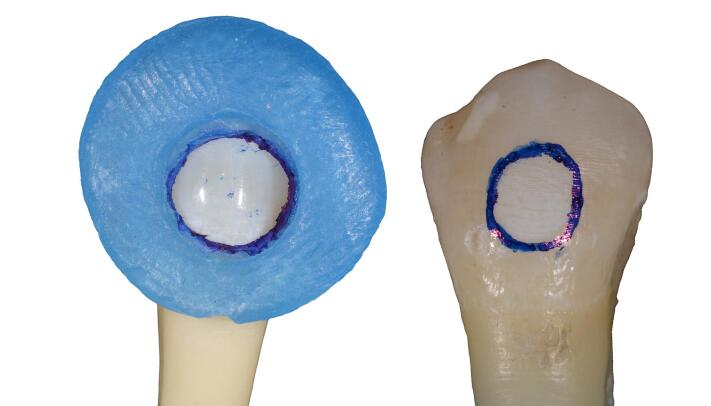
Blue wax mold used to mark a repeatable circular area on every dental element, as shown on the right

 A caries-free, fracture-free, and demineralization-free surface was selected for each sample, and a 3-mm circular area was marked using a permanent marker and a mold made of soft blue wax (Zeta Zingardi, Industria Zingardi srl, Novi Ligure, Italy) (Figure 1).

**Figure 2:**
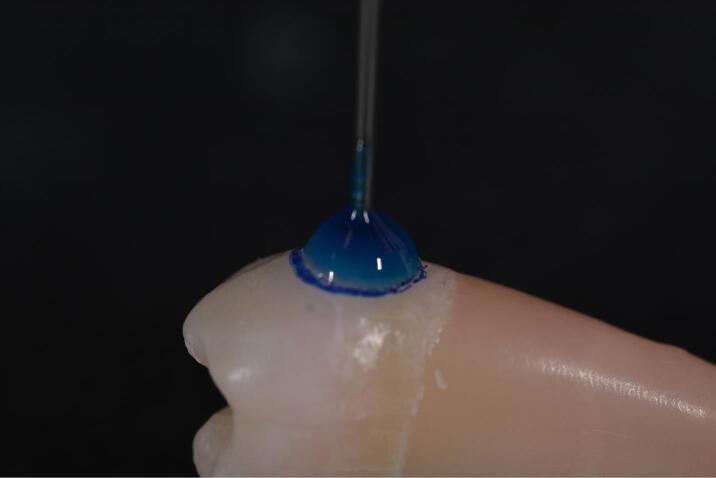
Application of 37% orthophosphoric acid on the pre-marked area of each dental element

**Figure 3:**
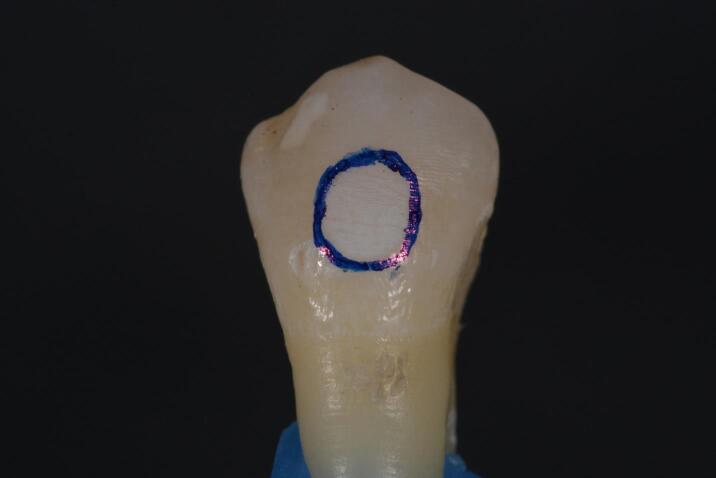
Example of an in vitro white spot lesion

 To create a visually detectable demineralized area, 37% orthophosphoric acid (Scotchbond^TM^ Universal Etchant, 3M ESPE, 2015) was applied for 1 minute and then rinsed with water for 30 seconds (Figures 2 and 3).

 The samples were stored in saline solution. Each sample was assigned an alphanumeric code to record color measurements at different time intervals and facilitate subsequent comparisons. The samples were then randomly divided into two groups of 30 units each using Microsoft Excel software (Excel version 16.84, Microsoft Office, 2024).

- Group 1: Remineralization treatment with Mi Paste Plus© (GC Europe N.V.)
- Group 2: Remineralization treatment with Mi Paste Plus© (GC Europe N.V.) + application of Scotchbond Multi-Purpose© adhesive (3M ESPE 2015)

**Figure 4:**
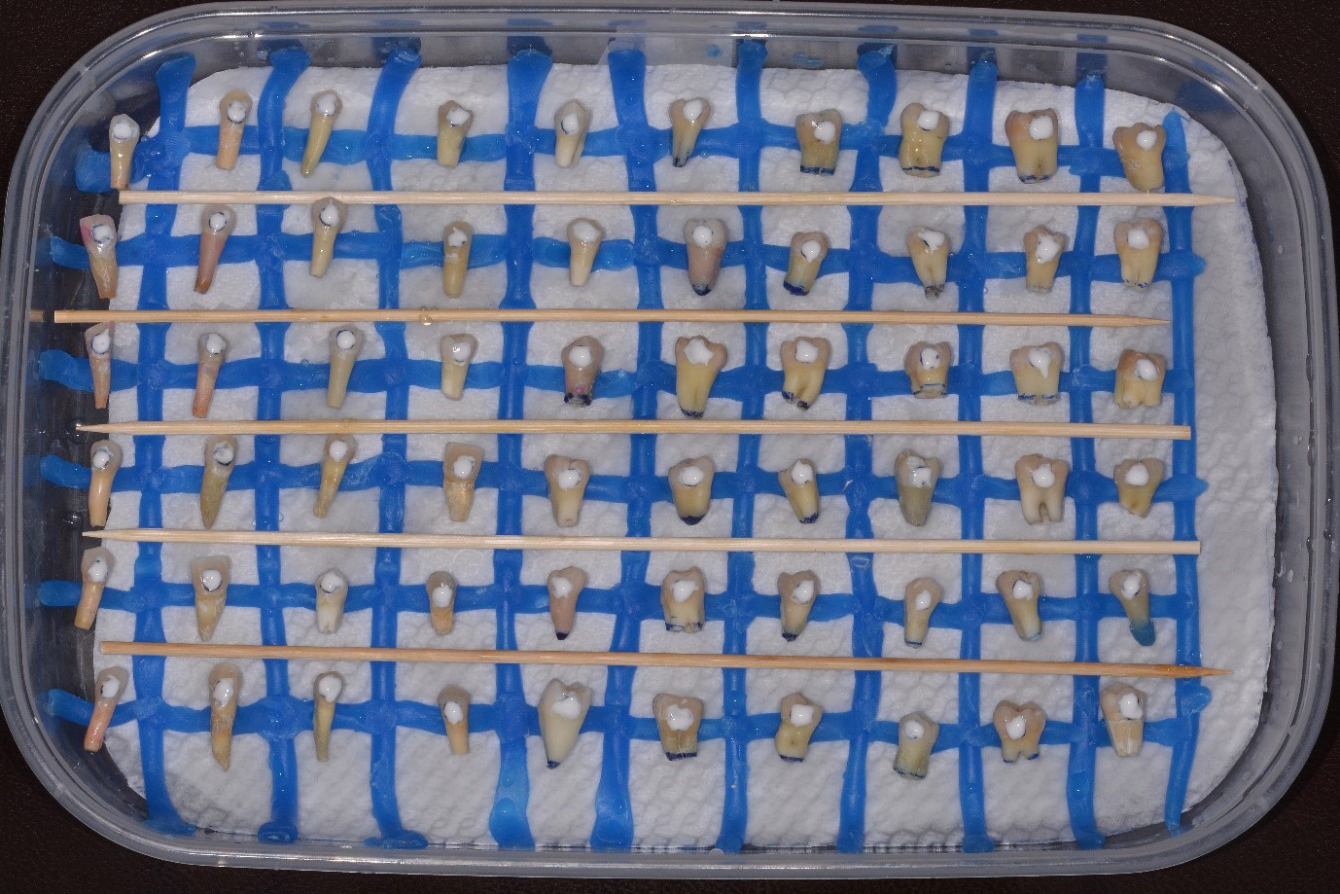
A container used to store the dental elements during the remineralization process

**Figure 5:**
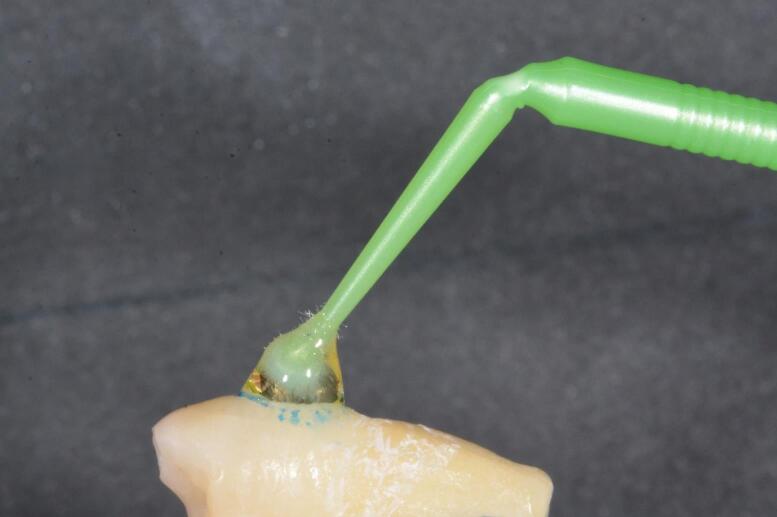
Application of Scotchbond Multi-Purpose© Adhesive with a microbrush for 1 minute on the marked area

The two groups were subjected to different treatments, specifically:

 1. Group 1 (Mi Paste Plus©)

 a) Application of an appropriate layer of Mi Paste Plus© to the previously delimited area using a microbrush

 b) Storage of the samples in a humidified container (Figure 4) layered with a damp cloth on the bottom and blue wax strips to keep the dental elements stable and organized, which simulated the moist environment of the oral cavity to prevent dehydration of the teeth

 c) After 6 hours, the samples were rinsed with water for 10 seconds to remove the remineralizing material

 d) Storage in physiological saline solution

 e) The procedure was repeated every day for 14 days, as recommended by the manufacturer and supported by literature.^15,16^

 2. Group 2 (Mi Paste Plus© + Scotchbond Multi-Purpose©)

 a) The remineralization treatment was performed as described for group 1 (steps a, b, and c)

 b) Application of Scotchbond Multi-Purpose© Primer using a microbrush for 3 minutes

 c) Evaporation of excess primer with a gentle stream of compressed air

 d) Application of Scotchbond Multi-Purpose© Adhesive (3M ESPE 2015) using a microbrush for 1 minute (Figure 5)

 e) Photopolymerization for 40 seconds

 f) Storage of the samples in physiological saline solution for 48 hours to allow for rehydration.

**Figure 6:**
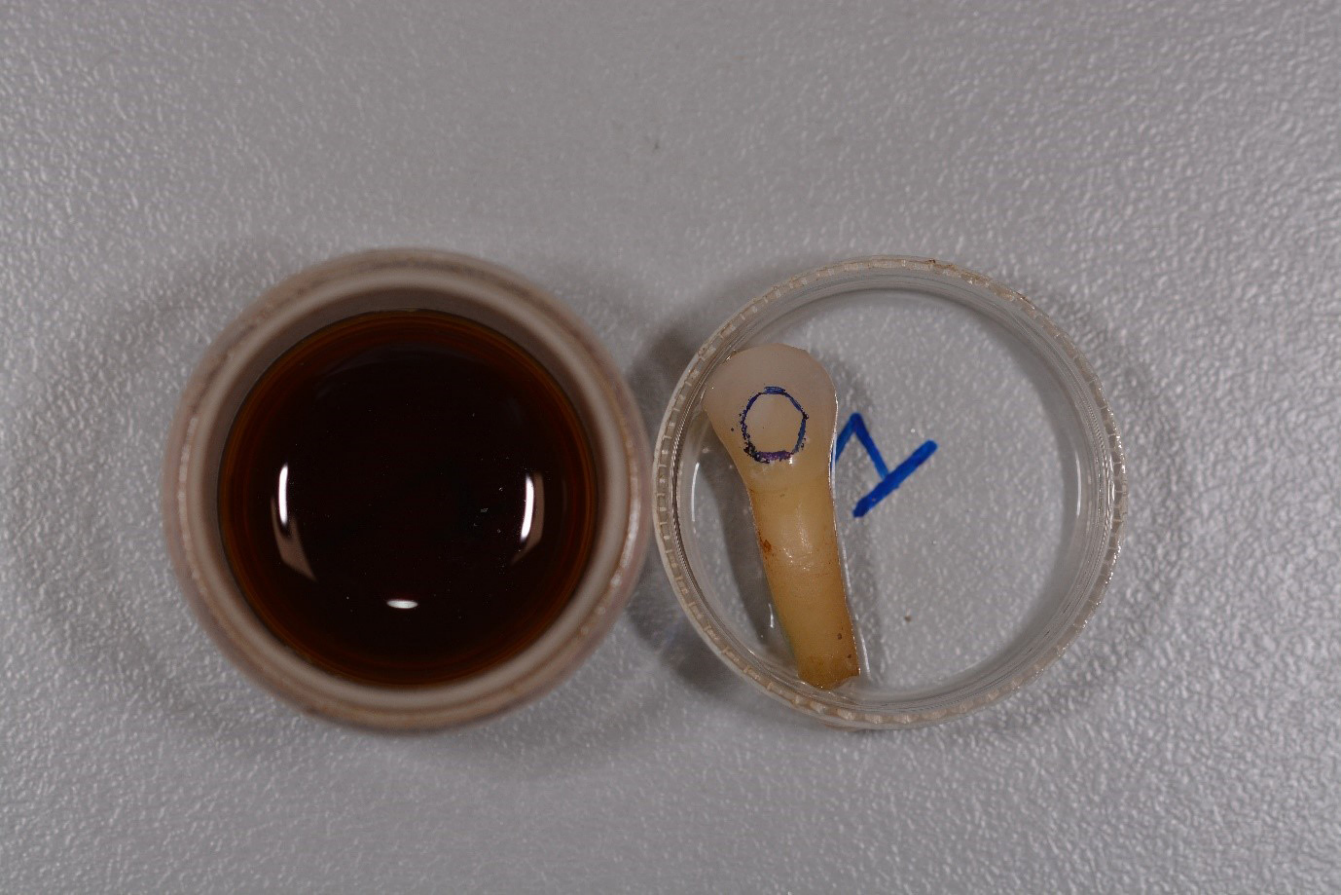
Instant coffee solution used to immerse the dental elements

**Figure 7:**
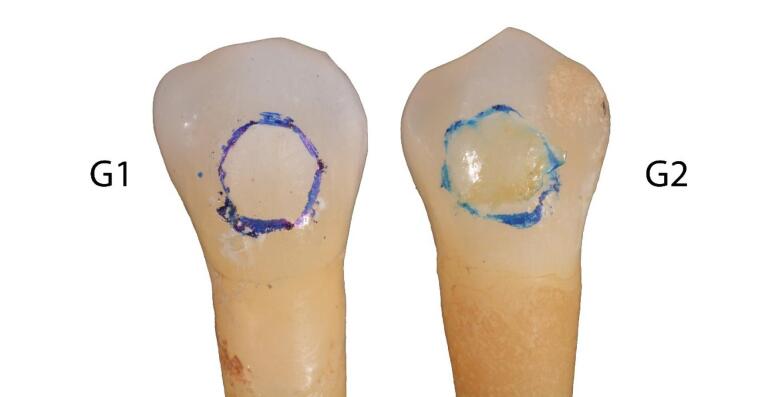
Example of one dental element from group 1 and one from group 2 after immersion in instant coffee solution

 Once properly rehydrated, the samples in both groups were immersed in coffee for 24 hours (Figures 6 and 7).

 The instant coffee used, and the solution was prepared by dissolving 3.6 g of coffee in 300 mL of distilled water, which had been previously boiled.^17^ The samples were then removed from the solution and rinsed with water for 20 seconds.

###  Color evaluation 

 Color evaluation was performed on spectrophotometric images of all samples from both groups at different time intervals:

- T1: After the creation of the WSL
- T2: At the end of the remineralization treatment
- TR: After applying the adhesive layer (only for group 2)
- T3: After 24 hours of immersion in coffee

Before each color evaluation, the samples were allowed to rehydrate in physiological saline solution for 48 hours.

 A condensation silicone support (Zetalabor Putty Hard, Zhermack Dental, Germany) was created for color recording of each tooth. Three wells were made in the silicone before it set. Two reference teeth were placed in the lateral wells to standardize all measurements, ensuring consistent results. The sample to be analyzed was placed in the central well.

**Figure 8:**
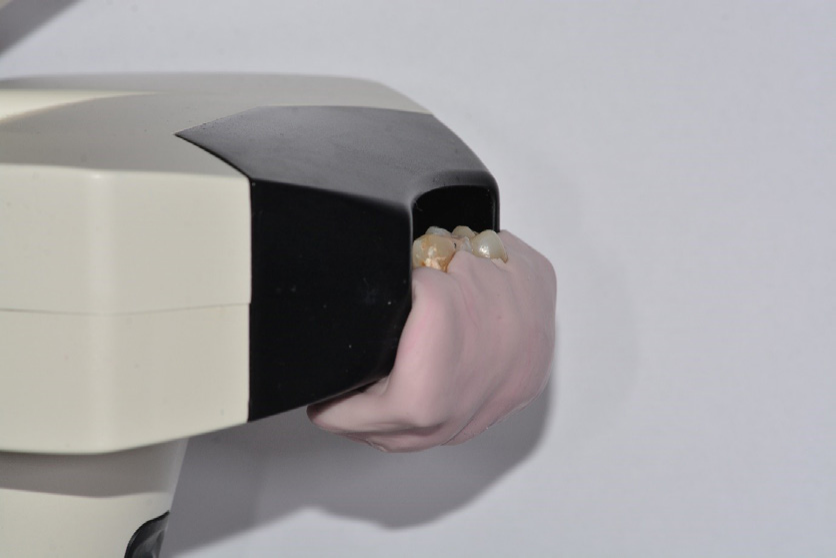
Pink silicone support molded around the spectrophotometer sensor to simulate the gingiva

 The silicone used was pink in color and was molded with a scalloped design to simulate the gingiva. This support was necessary because the spectrophotometer’s color detection is influenced by adjacent elements and surrounding tissues (Figure 8).

**Figure 9:**
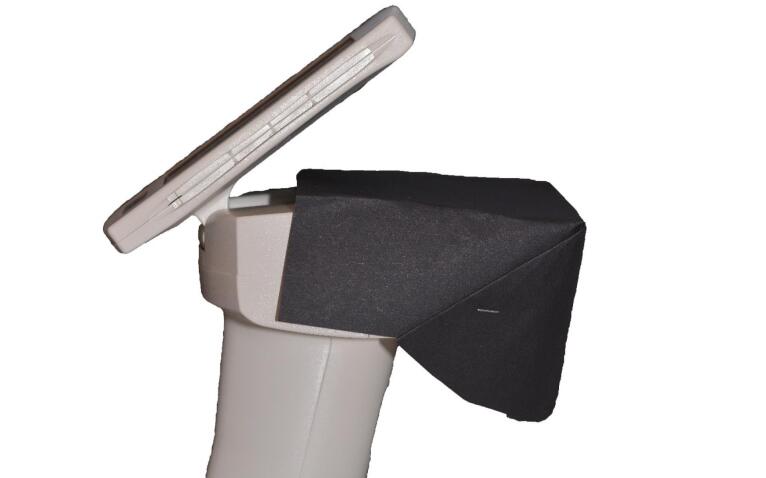
Black cardboard applied around the spectrophotometer sensor

 An additional standardization of the measurements was achieved by shaping the silicone support to position it near the spectrophotometer sensor in a repeatable manner for all measurements. During the image recording process, a black cardboard was placed around the support to simulate the dark environment of the oral cavity (Figure 9).

 Once acquired, the images were downloaded to a computer via the appropriate USB cable and imported into the “MHT SpectroShade” software for storage.

 To evaluate the mimetism and color stability of the treated area compared to the healthy tooth, the L*a*b* values of the two treatments were obtained via the spectrophotometer to calculate ΔE. In particular, using the MHT SpectroShade software, two points were selected, designated as P1 and P2. Point P1 was located within the treated area, while point P2 was located on the dental surface surrounding the treated area.

 These two points were reproduced in the same area in the images captured at the various time intervals (T_1_, T_2_, T_R_, and T_3_). To do this, the circular selection tool provided by the software was used, ensuring that the size of points P1 and P2 remained consistent in each recording. To ensure the repeatable positioning of P1 and P2 across images, their X and Y coordinates on the dental element were identified and recorded using a digital ruler overlaid on the images (ScreenRuler v.0.10.0, Bluegrams).

 Before each evaluation, the spectrophotometer was calibrated according to the manufacturer’s instructions. After each measurement, the samples were returned to the physiological saline solution. Once the L*a*b* values for points P1 and P2 were collected at each time interval, ΔE values were calculated as follows:


$$dE*=\sqrt{dL^{*2}+da^{*2}+db^{*2}}$$

The ΔE values obtained were:

- ΔET1 (G1 and G2)
- ΔET2 (G1 and G2)
- ΔETR (G2)
- ΔET3 (G1 and G2)

**Figure 10:**
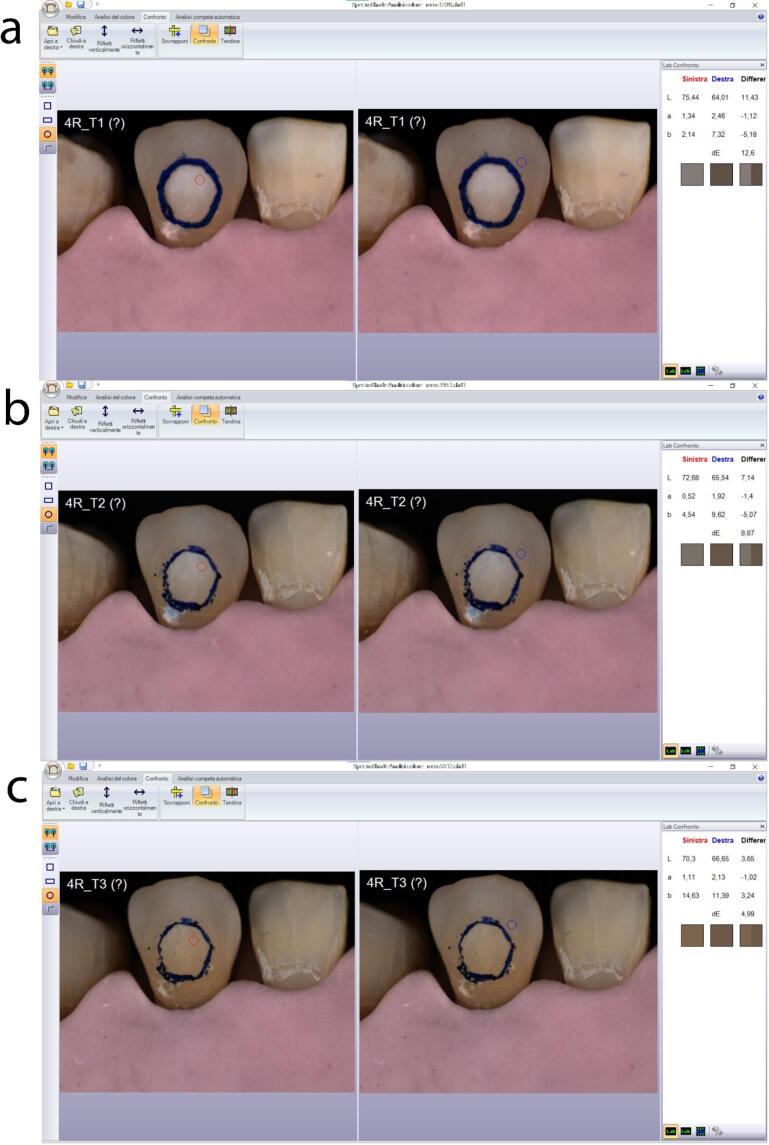
Screenshots from the “MHT SpectroShade” software used for confrontations at different time points; red and blue circles are the two points selected inside and outside the marked area in a repeatable way by using the digital ruler. (a) Confrontation of the two points on the same dental element from group 1 at T_2_ (after the creation of the in vitro white spot). (b) Confrontation of the two points on the same dental element from group 1 at T_2_ (after remineralization). (c) Confrontation of the two points on the same dental element from group 1 at T_3_ (after immersion in instant coffee solution)

**Figure 11:**
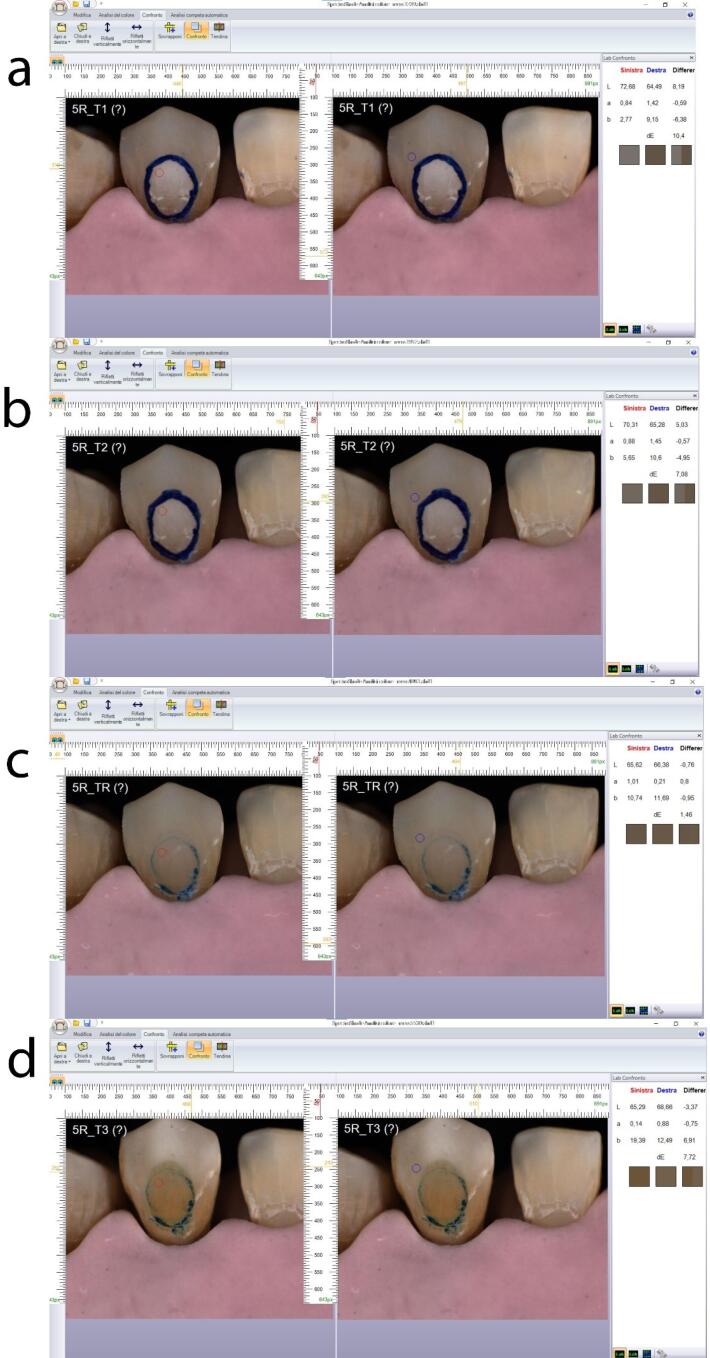
Screenshots from the “MHT SpectroShade” software used for confrontations at different time points; red and blue circles are the two points selected inside and outside the marked area in a repeatable way by using the digital ruler. (a) Confrontation of the two points on the same dental element from group 2 at T_1_ (after the creation of the in vitro white spot). (b) Confrontation of the two points on the same dental element from group 2 at T_2_ (after remineralization). (c) Confrontation of the two points on the same dental element from group 2 at T_R_ (after primer and bonding application). (d) Confrontation of the two points on the same dental element from group 2 at T_3_ (after immersion in instant coffee solution)

In the following images (Figures 10 and 11), two examples of samples from groups 1 and 2 are shown.

 The images were taken at T_1_, T_2_, and T_3_ for group 1, and at T_1_, T_2_, T_R_, and T_3_ for group 2. In both images, the red circle represents point P1 (reference for the color of the treated area), and the blue circle represents point P2 (reference for the color of the surrounding surface to the treated area). On the right, the comparison of L*a*b* values between points P1 and P2 and the corresponding ΔE can be observed.

###  Statistical analysis 

 One sample from each group was excluded from the study due to a color change during the experiment, which compromised its integrity.

 The data were analyzed using the statistical software STATA 17 (StataCorp, 1985, California, USA). All tests were considered statistically significant with a *P* value ≤ 0.05.

 The data did not meet the criteria required for the use of parametric tests, so non-parametric tests were employed for all analyses.

###  Descriptive analysis 

 The median and interquartile range of the ΔE values for both groups were calculated at the different time intervals.

 A graphical representation of the ΔE trends was created for each group at all time intervals.

###  Intergroup analysis 

 The intergroup analysis was conducted as follows:

- Evaluation of the mimetism between the samples of G1 and G2 (the remineralization treatment only vs. the remineralization treatment with resin) using the Wilcoxon Mann-Whitney statistical test, comparing ΔET2_G1_ with ΔETR_G2_.
- Evaluation of the stability of the two treatments when the sample was exposed to a pigmenting substance using the Wilcoxon Mann-Whitney statistical test, comparing ΔET3_G1_ with ΔET3_G2_.

####  Intragroup analysis 

 The intragroup analysis was performed using the paired Wilcoxon Mann-Whitney test to assess the stability of the result for each treatment after immersion in the pigmenting substance, comparing the following values:

- G1: ΔET2 vs. ΔET3
- G2: ΔETR vs. ΔET3

## Results

Tables 1 and 2 show the ΔE values calculated for both groups at the different time intervals:

- T1: after the creation of the WSL
- T2: at the end of the remineralization treatment
- TR: after the application of the adhesive layer (only for group 2)
- T3: after 24 hours of immersion in coffee

**Table 1:** ΔE values for samples of group 1 at T_1_, T_2_, and T_3_

**Group 1 (MiPaste Plus©)**	**ΔET1**	**ΔET2**	**ΔET3**
R1	10.2	7.11	6.49
R4	12.6	8.87	4.99
R8	9.45	9.13	2.28
R10	11.47	9.23	4.91
R11	4.57	3.9	2.38
R12	4.56	4.5	6.23
R15	2.72	1.91	2.03
R16	12.39	8.54	2.1
R17	7.18	6.15	5.23
R18	4.66	3.94	5.51
R19	5.01	3.17	3.12
R21	2.32	2.34	4.04
R22	2.5	2.1	4.69
R26	8.66	7.85	4.16
B1	10.19	9.18	7.57
B3	7.07	5.32	2.27
B4	6.51	6.61	1.91
B5	5.43	5.2	5.41
B8	4.48	1.01	7.11
B16	8.1	5.88	3.83
B17	8.29	6.11	3.69
B18	4.71	3.61	5.58
B19	7.22	1.39	1.01
B25	6.76	5.17	1.83
B27	10.35	7.48	3.64
B29	7.61	5.94	4.7
B30	6.49	4.84	3.09
B31	10.89	9.26	5.48
B33	4.97	3.46	4.21

**Table 2:** ΔE values for samples of group 2 at T_1_, T_2_, T_R_, and T_3_

**Group 2 (MiPaste Plus©+Scotchbond Multi.-Purpose©)**	**ΔET1**	**ΔET2**	**ΔETR**	**ΔET3**
R2	9.58	5.8	1.96	3.33
R3	7.53	5.17	2.48	5.56
R5	10.4	7.08	1.46	7.72
R7	11.75	9.82	3.65	4.64
R9	8.34	6.23	3.88	4.66
R13	11.86	8.95	2.44	10.78
R14	3.55	3.52	2.88	2.64
R20	5.66	4.59	3.33	8.43
R23	5.91	5.99	2.97	4.85
R24	7.88	5.72	2.14	6.56
R25	8.25	5.35	2.53	7.5
B2	7.52	5.61	2.79	7.97
B6	5.11	2.24	1.49	9.71
B7	10.55	7.46	3.92	10.82
B9	6.19	5.03	3.03	4.17
B10	7.46	6.71	3.4	12.08
B11	8.93	4.9	2.73	6
B12	15.56	13.95	2.61	7.49
B13	6.79	4.02	2.01	9.67
B14	5.1	5.3	3.21	13.5
B15	7.11	4.1	3.49	9.18
B21	7.54	7.15	2.86	9.98
B22	8.34	5.15	3.08	3.8
B23	11.33	8.53	1.36	7.21
B24	12.29	11.21	2.47	11.62
B26	5.75	5.37	4.57	3.53
B28	4.59	3.42	2.54	4.38
B32	2.15	2.01	1.56	3.3
B34	4.23	3.5	1.33	6.5

**Figure 12:**
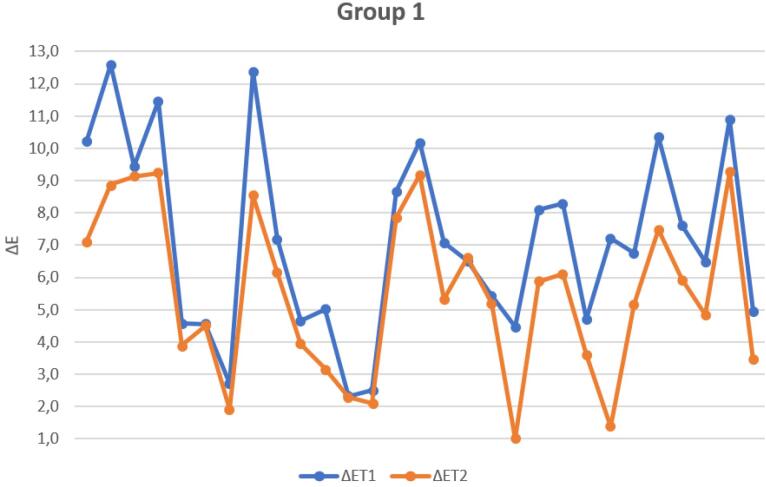
ΔE values at T_1_ and T_2_ for the samples of group 1

 The graph (Figure 12) below shows the ΔE values at T_1_ and T_2_ for the samples in G1, illustrating the extent of the change after the remineralization treatment.

**Figure 13:**
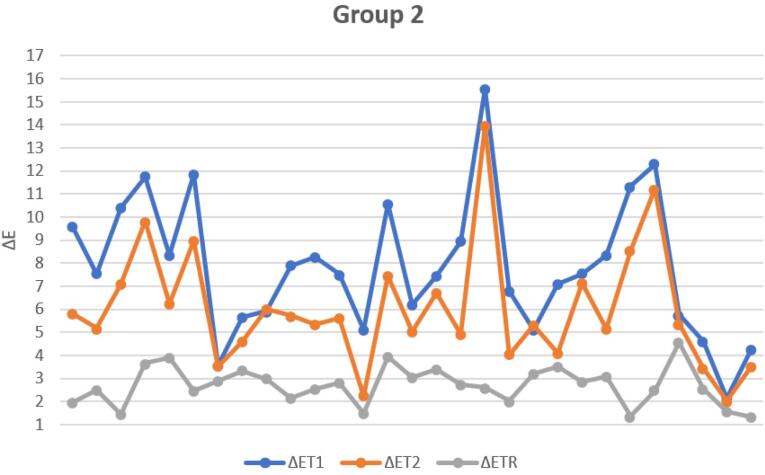
ΔE values at T_1_, T_2_, and T_R_ for the samples of group 2

 The graph (Figure 13) below shows the ΔE values at T_1_, T_2_, and T_R_ for the samples in G2, illustrating the extent of the change after the remineralization treatment and the application of the adhesive layer.

###  Intergroup analysis 

####  ΔET2G1 vs. ΔETRG2 

Table 3 shows the median and interquartile range of ΔET2 and ΔETR for G1 and G2, respectively.

**Table 3:** Median and interquartile range of ΔET2 for group 1 and ΔETR for group 2

**Group**	**Median**	**Q1**	**Q3**
G1	5.94	3.61	7.48
G2	2.73	2.17	3.21

**Figure 14:**
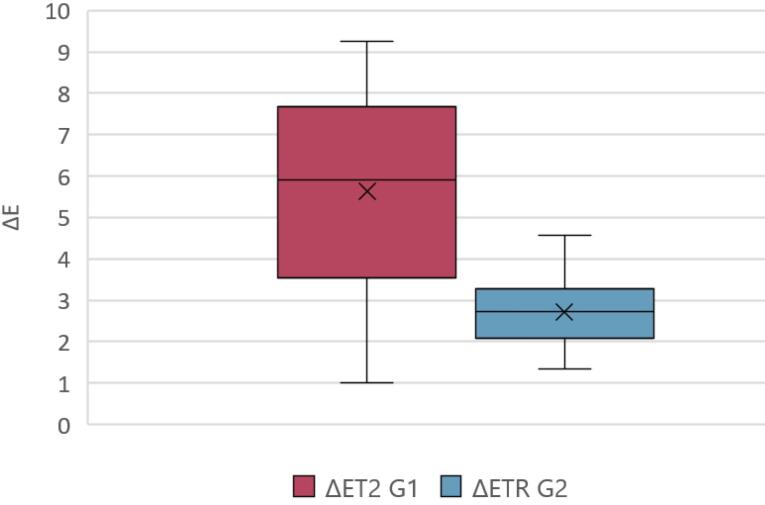
Box-plot of ΔET2 (group 1) and ΔETR (group 2)

 The graph below (Figure 14) illustrates the median values and interquartile ranges of ΔET2 and ΔETR in a box-and-whisker plot.

 Statistical analysis using the Wilcoxon Mann-Whitney test revealed a statistically significant difference (*P* = 0.0001) between G1 and G2 regarding the mimetic capacity of the two treatments.

####  ΔET3G1 vs. ΔET3G2 

Table 4 presents the medians and interquartile ranges of ΔET3 for both groups.

**Table 4:** Median and interquartile range for ΔE values for group 1 and group 2 at T_3_

**Group**	**Median**	**Q1**	**Q3**
G1	4.16	2.38	5.41
G2	7.21	4.64	9.67

**Figure 15:**
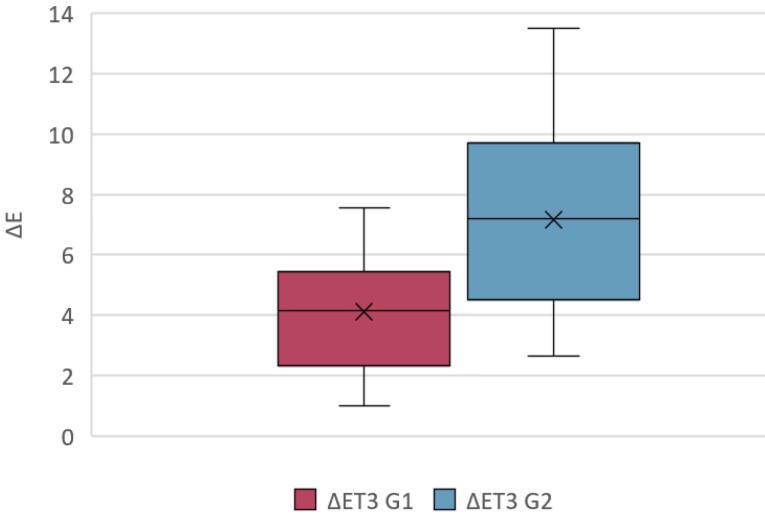
Box plot of ΔET3 values for groups 1 and 2

 The graph below (Figure 15) presents the median values and interquartile ranges of ΔET3 for G1 and G2 in a box-and-whiskers plot.

 The Wilcoxon Mann-Whitney test revealed a statistically significant difference (*P* = 0.0002) between G1 and G2 concerning the color stability of the two treatments over time.

###  Intragroup analysis

####  G1: ΔET2 vs. ΔET3

Table 5 presents the medians and interquartile ranges of ΔET2 and ΔET3 for the samples in G1.

**Table 5:** Median and interquartile range for ΔE values of group 1 at T_2_ and T_3_

**ΔE**	**Median**	**Q1**	**Q3**
ΔE T2	5.94	3.61	7.48
ΔE T3	4.16	2.38	5.41

**Figure 16:**
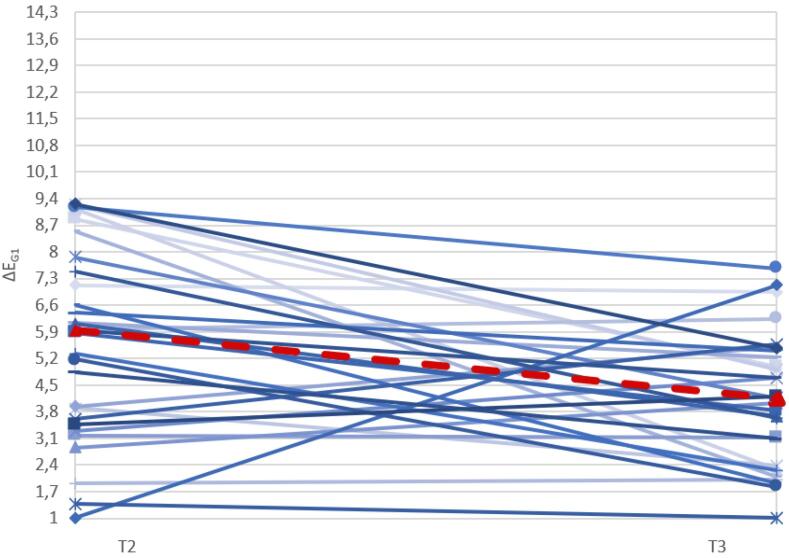
ΔE values for group 1 before and after immersion in the staining solution (T_2_ and T_3_). Each line in shades of blue represents the trend of ΔE for each sample in group 1 between T_2_ and T_3_. The red line represents the trend of the mean value between the two time points

 The graph below (Figure 16) displays the ΔE values of the G1 samples before and after exposure to the pigmenting substance (T2 and T3, showing a modest variation in ΔE between the samples before and after the pigmenting treatment, with the median decreasing after treatment.

 Statistical analysis using the Wilcoxon paired test showed a statistically significant difference (*P* = 0.005) in ΔE_G1_ before and after exposure to the pigmenting substance.

####  G2: ΔETR vs. ΔET3

Table 6 shows the medians and interquartile ranges of ΔETR and ΔET3 for the samples in G2.

**Table 6:** Median and interquartile range for ΔE values of group 2 at T_R_ and T_3_

**ΔE**	**Median**	**Q1**	**Q3**
ΔE TR	2.73	2.14	3.21
ΔE T3	7.21	4.64	9.67

**Figure 17:**
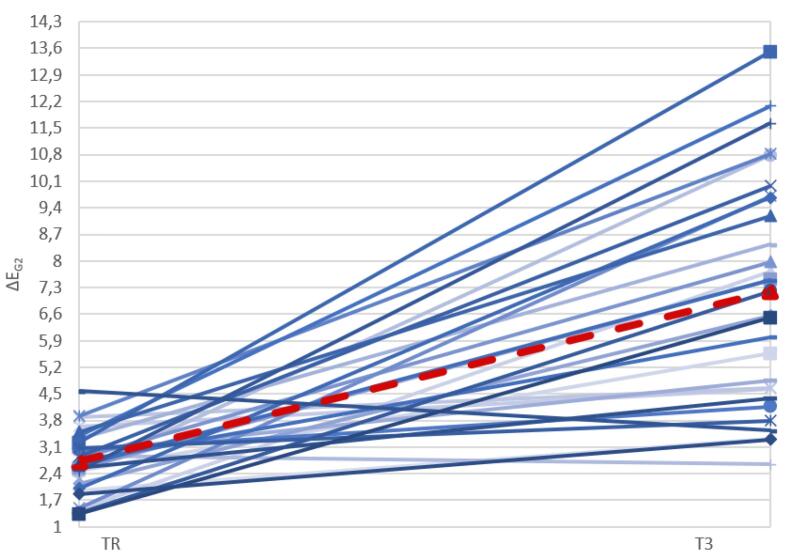
ΔE values for group 2 before and after immersion in the staining solution (T_R_ and T_3_). Each line in shades of blue represents the trend of ΔE for each sample in group 2 between T_R_ and T_3_. The red line represents the trend of the mean value between the two time points

 The graph below (Figure 17) shows the ΔE values of the G2 samples before and after exposure to the pigmenting substance, with a marked variation in ΔE between the sample before and after the pigmenting treatment, with the median increasing after treatment.

 Statistical analysis using the Wilcoxon paired test revealed a statistically significant difference (*P* = 0.0000) in ΔE_G2_ before and after exposure to the pigmenting substance.

## Discussion

 WSLs are aesthetic-impact lesions localized primarily on the vestibular surface of anterior teeth, caused by enamel demineralization.^16^ Their reversible nature justifies treatment approaches that aim to restore both structure and appearance.

 This study investigated the optical effectiveness of a remineralizing agent (Mi Paste Plus©), used alone or in combination with an adhesive system (Scotchbond Multi-Purpose©), with a focus on color changes rather than structural enamel modifications. Objective color measurements were obtained using the MHT SpectroShade Micro spectrophotometer (MHT Srl, Oxnard, CA, USA),^18^ which, although primarily intended for anterior sectors, was successfully used on posterior teeth in an in vitro setting.

 Clinically acceptable color differences were defined as ΔE values < 3.3 as stated in recent literature.^19-22^ Only the group treated with the combined protocol (G2) achieved values within this threshold (median ΔE = 2.73; IQR: 2.17–3.21), whereas the mousse alone (G1) yielded higher, clinically unacceptable values (median ΔE = 5.94; IQR: 3.61–7.48). The difference between the two groups was statistically significant (Mann–Whitney U test, P = 0.0001). These findings support the existing literature, which suggests that adhesives can enhance WSL appearance by reducing light scattering, thanks to their refractive index similarity to natural enamel.^11,13,23^ The resin component fills superficial microporosities, equalizes the optical pathway, and consequently increases translucency. This is also true for specific resin-based agents, such as Icon, used for treating WSLs, which, due to their refractive index similar to that of hydroxyapatite, provide a mimetic appearance to the treated WSLs.^7^

 Nevertheless, certain authors have shown that adhesive resins alone may be insufficient, especially in deeper lesions.^13,24^ The present study supports emerging evidence that a preceding remineralization step, such as with CPP-ACFP-containing products, can improve the optical performance of subsequently applied resin.^25^

 Reducing the depth of demineralization may enhance the effectiveness of resins with limited penetration capacity, such as those used in adhesive systems, by allowing better infiltration into the lesion. To speculate on this hypothesis, studies on the enamel structure before and after remineralization would be necessary, a topic that was not the focus of this study.

 Using the adhesive system offers another advantage: reduced costs for the practitioner. Every dental practice has at least one adhesive system for conservative and restorative procedures; therefore, achieving an aesthetic mimetic result for white spots could be accomplished without additional costs by using this type of resin.

 Marked variability was observed in the ΔE values of G1 compared to those of G2, which is evident in the data dispersion illustrated in Figures 12 and 13. In both graphs, there is significant variability in the aesthetic outcome of the remineralizing treatment, regardless of the initial condition of the treatment area. Some samples demonstrated a significant improvement, with ΔE values decreasing from greater than 3.3 to a clinically acceptable level, as defined by the threshold values considered in this study. On the other hand, other samples remained stable or showed only slight improvement after treatment. By contrast, the adhesive group displayed a tighter distribution of ΔE, indicating more predictable outcomes.

 Several factors may underlie the high variance obtained with the mousse, including treatment duration, method of WSL creation, and the absence of saliva.^26,27^

 In our in vitro protocol, the mousse was applied for 6 h a day over 14 days, consistent with the manufacturer’s instructions; nonetheless, a longer application period might enhance results.^16^ This aligns with a study by Bröchner et al,^28^ which highlighted that the efficacy of CPP-ACP depends on the duration of the treatment.

 In most in vitro studies in the literature, artificial WSLs are obtained using a demineralizing solution that leads to the formation of a subsurface demineralization zone. This is essential for allowing an adequate evaluation of the surface microhardness of the samples and variations in enamel structure following different remineralizing treatments. In this experiment, artificial lesions were produced using 37% phosphoric acid for 60 s, resulting in a predominantly superficial demineralization zone rather than the subsurface pattern usually found in vivo; this could limit the diffusion of calcium-phosphate complexes, attenuating optical improvement.^29^ This methodological choice was motivated by the study’s aim to evaluate only the aesthetic performance of the two treatments, which required the demineralized area to be visible to the naked eye initially to identify optical changes. This could have influenced the remineralizing activity of the CPP-ACFP-based mousse. The calcium and phosphate molecules released by the bioactive substance are capable of filling the porosities in the layers beneath the intact enamel surface, typical of natural WSL.^16^ The persistence of a demineralized surface could thus have affected the aesthetic outcome of the remineralizing treatment.

 The presence of saliva significantly enhances the efficacy of CPP-ACFP because salivary proteins and peptides bind to the complex, enhancing its ability to transport calcium and phosphate ions into the enamel and facilitating the repair of demineralized areas.^26^ This could be one of the reasons why the results of in vitro studies like this one or those by Yadav et al^30^ and Yuan et al,^31^ which do not benefit from the action of saliva, have not shown a remarkable effect of CPP-ACP on the color restoration of a WSL. In contrast, the in vivo study by Simon et al.^32^ reported a significant improvement of the WSL following treatment with CPP-ACP. It could be interesting to conduct the experiment using artificial saliva in combination with the remineralizing treatment to more closely simulate in vivo conditions.

 The second aim of this study was to investigate the long-term color stability of the two treatments when exposed to a pigmenting substance. No study to date has compared the color stability of the remineralizing treatment with that of the remineralizing treatment and adhesive layer combined.

 After 24 h of immersion in coffee, equivalent to roughly one month of intraoral exposure,^17^ both groups exhibited ΔE values exceeding 3.3; however, median ΔE rose to 7.21 (IQR 4.64–9.67) in group 2 versus 4.16 (IQR 2.38–5.41) in group 1 (Wilcoxon’s signed-rank test, *P* = 0.0002). This confirms the intrinsic and extrinsic propensity of resin materials to absorb pigments,^33,34^ particularly from beverages such as coffee that show chemical affinity for the polymer matrix.^35^ The greater scatter observed for group T_R_ reflects the wider range of initial colors and the resin’s differential susceptibility to staining.^33,34^ In this study, the samples were intentionally immersed in a coffee solution because the literature has demonstrated this beverage’s tendency to alter the color of resin materials due to the chemical compatibility between the beverage and the polymers in the resin.^35^

 This could explain why the samples treated with resin underwent a greater color change compared to the group treated only with the remineralizing mousse.

 Technically, effective masking of a WSL requires both a reduction in the L* factor and an increase in the b* factor.^23^ Coffee produces a yellowish surface deposit, increasing b* and thereby diminishing ΔE in the remineralization-only specimens, which could explain why the ΔE of G1 after pigmentation approached clinically more acceptable values compared to those obtained after the remineralizing treatment. However, b* was not separately analyzed here; thus, future studies should quantify its contribution and evaluate different staining substances as well as extended immersion times, which could also reveal different patterns of discolouration.^32,36^

## Conclusion

 The combined use of CPP-ACFP and an adhesive system yielded better results in terms of ΔE compared to the remineralizing treatment alone, which was performed over 14 days.

 The application of an adhesive system can be considered a valid option to improve the aesthetic outcome following a remineralizing treatment. However, it is essential to note that this approach carries a risk of resin pigmentation, which may necessitate additional treatments or increased attention to diet and oral hygiene to maintain aesthetic results over time.

 These findings underscore the importance of balancing immediate aesthetic gain with long-term color stability, highlighting the need for protocols that integrate remineralization and sealing, as well as maintenance strategies, under clinically realistic conditions.

## Competing Interests

 The authors declare no relevant financial or non-financial interests.

## Data Availability Statement

 The datasets analyzed during the current study are available from the corresponding author on reasonable request.

## Ethical Approval

 The study was performed per the ethical standards as laid down in the 1964 Declaration of Helsinki and approved by the Ethics Committee of South Est Veneto (approval number: 430CET).

## Informed Consent

 Informed consent (including for publication) was obtained from all individual participants who donated dental elements for this research.
